# Initial experience of clinical implementation of an Online Simulation‐free CBCT‐based Adaptive Radiotherapy (OSCAR) workflow for palliative and urgent treatment indications

**DOI:** 10.1002/acm2.70727

**Published:** 2026-08-03

**Authors:** Robert Schindhelm, Anne Richter, Philip Kleine, Jörg Tamihardja, Victor Lewitzki, Bülent Polat, Marcus Zimmermann, Frederick Mantel, Thomas Fischer, Sebastian Heß, Ingulf Lawrenz, Franziska Künzig, Felix Schilling, Andrea Wittig‐Sauerwein, Gary Razinskas

**Affiliations:** ^1^ Department of Radiotherapy and Radiation Oncology University Hospital Würzburg Würzburg Germany

**Keywords:** cone‐beam CT, Ethos, online‐adaptive radiotherapy, simulation‐free workflow

## Abstract

**Background:**

The current standard radiotherapy treatment planning workflow typically requires several hours to days, during which patients must wait before treatment can be initiated. In certain oncological situations, however, treatment should commence as soon as possible. This is particularly critical in oncological emergencies to prevent further complications, as well as in palliative treatment indications where rapid symptom relief is essential.

**Purpose:**

In this study, we report our initial clinical experience with the online simulation‐free CBCT‐based adaptive radiotherapy (OSCAR) workflow, focusing on feasibility, workflow timing, and dosimetric plan quality.

**Methods:**

Seven anatomical regions were predefined for rapid treatment initiation using standardized RT intent templates in Ethos (Varian Medical Systems) with HyperSight CBCT. The OSCAR workflow was implemented including institutional safety strategies and alternative workflows where necessary. For the first patients treated with OSCAR, workflow timing was assessed, post‐adaptive treatment strategies were documented, and Ethos on‐couch IMRT plans were compared dosimetrically to corresponding IGRT VMAT plans generated in Eclipse on the HyperSight CBCT.

**Results:**

The OSCAR workflow was successfully established for the first 11 patients requiring rapid initiation of radiotherapy. The average total time required to initiate treatment was approximately 1 h and 50 min, while the adaptive treatment fraction (including patient setup, online adaptation, and irradiation) required approximately 20 min. The dosimetric comparison between static gantry IMRT and VMAT plans demonstrated that both techniques met clinical acceptance criteria for target coverage and OAR sparing.

**Conclusions:**

The OSCAR workflow presented here enables rapid and clinically feasible treatment initiation without the need for a dedicated planning CT by combining standardized RT intent templates with HyperSight‐CBCT‐based online adaptation. While evaluated in a limited initial cohort and heterogeneous indications, the workflow demonstrated practicability and clinically acceptable plan quality. Future studies should evaluate scalability to additional disease sites and fractionation schemes, including ultra‐hypofractionated regimens.

## 1 INTRODUCTION

After the decision to proceed with radiotherapy and obtaining informed consent, one of the initial steps in the radiotherapy process chain is treatment planning, which includes the acquisition of a computed tomography (CT) simulation. This step is typically performed on a CT simulator and includes patient immobilization in the intended treatment position, as well as acquisition of electron density information in the form of Hounsfield units (HU), which is required for treatment planning and dose calculation. Subsequent workflow steps include, among others, contouring of target volumes, treatment prescription, and plan‐specific quality assurance (PSQA). The entire radiotherapy treatment planning process generally requires several hours to days, during which patients must wait before treatment can be initiated. Particularly for palliative indications, where patients may experience severe pain, or in oncological emergencies such as obstruction or compression of hallow organs, tumor‐related bleeding, or spinal cord compromise, treatment should start as soon as possible to prevent further complications and to achieve rapid relief of symptoms.^1^

In recent years, several approaches have been proposed to shorten time‐to‐treatment by reducing or eliminating individual steps of the conventional workflow. With the implementation of online adaptive radiotherapy (oART) and the ability to perform dose calculation directly on acquired cone‐beam CT (CBCT) images, treatment initiation can be substantially accelerated. Recent studies have introduced simulation‐free workflows that utilize diagnostic CT (dCT) data for treatment planning and contouring, thereby omitting the dedicated planning CT simulation. These approaches have demonstrated feasibility particularly in palliative and urgent clinical scenarios.^2^, ^3^, ^4^, ^5^ The Ethos system (Varian Medical Systems, Palo Alto, USA) integrates automated treatment planning and auto‐segmentation with high‐quality CBCT imaging, on which dose calculation can be performed directly.

However, clinical experience with fully online, simulation‐free workflows that rely exclusively on CBCT for on‐couch dose calculation remains limited. In particular, data on workflow feasibility, timing, and plan quality in a real‐world clinical environment are scarce. In this study, we report our initial clinical experience with the online simulation‐free CBCT‐based adaptive radiotherapy (OSCAR) workflow. The aim is to establish and evaluate the feasibility of this workflow for rapid treatment initiation in urgent and palliative indications, to analyze the associated workflow timing, and to assess the dosimetric quality of automatically generated adaptive static gantry IMRT plans in comparison to conventionally optimized VMAT plans.

## 2 METHODS

### 2.1 Treatment platform and imaging system

The online simulation‐free CBCT‐based adaptive radiotherapy (OSCAR) workflow was established on an Ethos system (Halcyon software version 4.0, Varian Medical Systems, Palo Alto, USA) equipped with the HyperSight kV‐imager. Among other features, this novel kV‐detector is characterized by an image acquisition time of approximately 6 s, improved image quality, and advanced Acuros iCBCT and Acuros iCBCT metal artifact reconstruction algorithms. These features provide superior image quality compared with other detectors, particularly regarding scatter correction, spatial resolution, contrast resolution, uniformity, noise, and HU accuracy.^6^, ^7^, ^8^ This enables plan optimization and dose calculation directly on the acquired HyperSight‐CBCT.

### 2.2 Clinical indications and patient selection criteria

**FIGURE 1:**
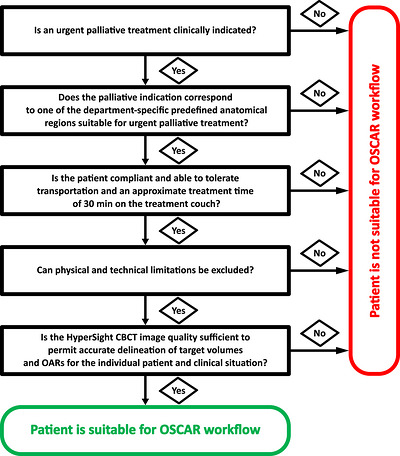
Individual steps for guiding the physician deciding whether the OSCAR workflow is applicable.

For the OSCAR workflow, clearly defined patient selection criteria are required to guide the physician in deciding whether the workflow can be initiated. The first step is to assess whether urgent palliative radiotherapy is indicated. Clinical indications include, for example, severe pain, bleeding, dyspnea, and neurological symptoms, for which prompt treatment initiation is generally indicated and may improve the patient's quality of life (QoL).^9^ The second step is to determine whether the treatment indication corresponds to one of the department‐specific predefined anatomical regions suitable for urgent palliative treatment. If these criteria are met, the next step is to assess whether the patient is compliant and able to tolerate both transportation and the approximate 30 min treatment time on the treatment couch. Thereafter, physical and technical limitations must be considered, including the dose that can be delivered to nearby critical organs as well as limitations of the Ethos treatment delivery unit, such as a maximum treatment field size of 28 × 28 cm^2^ for a single isocenter or 38.5 × 28 cm^2^ for two isocenters.^10^ Finally, it must be considered that target volumes and organs at risk (OARs) are contoured on the HyperSight CBCT rather than on a dedicated planning CT; therefore, the image quality must be sufficient to permit accurate delineation for the individual patient and clinical situation. Figure 1 illustrates the decision‐making workflow supporting physician selection of patients suitable for the OSCAR workflow.

The anatomical regions and indications suitable for the OSCAR workflow in our department are spinal metastases of the cervical, thoracic, and lumbar spine, as well as the sacrum, brain (e.g. acute neurological deficit), thorax (e.g. upper venous congestion), and cervix (e.g. acute cervical bleeding). For these indications, multiple fractionation schemes may be applied and different OARs must be considered. Table 1 summarizes the anatomical treatment sites, associated clinical indications, commonly used fractionation schemes, and the most relevant OARs considered for each anatomical region.

**TABLE 1:** Overview of anatomical regions, clinical indications, fractionation schemes, and relevant OARs defined within the RT intent templates for the OSCAR workflow.

Anatomical region and indication	Fractionation	Relevant organs at risk (OARs)
Cervical Spine e.g. tumor‐related acute or imminent spinal cord compression	10 × 3 Gy 5 × 4 Gy 14 × 2.5 Gy 1 × 8 Gy	Brainstem, Esophagus, Larynx, Oral Cavity, Parotids, Spinal Canal, Submandibular Glands, Thyroid, Trachea, Brachial Plexus
Thoracic Spine e.g. tumor‐related acute or imminent spinal cord compression	10 × 3 Gy 5 × 4 Gy 14 × 2.5 Gy 1 × 8 Gy	Brachial Plexus, Esophagus, Large Bowel, Liver, Lungs, Heart, Kidneys, Small Bowel, Spinal Canal, Spleen, Stomach, Thyroid, Trachea
Lumbar Spine e.g. tumor‐related acute or imminent spinal cord compression	10 × 3 Gy 5 × 4 Gy 14 × 2.5 Gy 1 × 8 Gy	Large Bowel, Kidneys, Liver, Small Bowel, Spinal Canal, Spleen, Stomach
Sacrum e.g. tumor‐related acute or imminent spinal cord compression	10 × 3 Gy 5 × 4 Gy 14 × 2.5 Gy 1 × 8 Gy	Femoral Heads, Large Bowel, Sigmoid Colon, Small Bowel, Spinal Canal
Brain e.g. acute neurological deficit such as vision impairment, impending herniation	10 × 3 Gy 5 × 4 Gy 14 × 2.5 Gy	Brainstem, Chiasm, Globes, Lacrimal Glands, Lenses, Oral Cavity, Optic Nerves, Parotids
Thorax e.g. upper venous congestion, tumor‐related obstruction of main bronchi	10 × 3 Gy 5 × 4 Gy 14 × 2.5 Gy 1 × 8 Gy	Brachial Plexus, Esophagus, Heart, Larynx, Lungs, Spinal Canal, Thyroid, Trachea
Cervix e.g. acute cervical bleeding	10 × 3 Gy 5 × 4 Gy 16 × 2.5 Gy	Bladder, Femoral Heads, Large Bowel, Rectum, Sigmoid Colon, Small Bowel, Spinal Canal

### 2.3 Reference imaging and dataset preparation

In the OSCAR workflow, a planning CT is not strictly required as a reference image; instead, the most recent diagnostic CT (dCT) or PET‐CT with identical axial in‐plane pixel spacing (i.e., identical horizontal and vertical pixel dimensions) can be used. If no dataset with identical in‐plane pixel spacing is available, the Ethos software cannot automatically generate the body contour and OARs. Notably, the CT dataset serves solely as an anatomical reference: As long as the treatment region has been imaged using another modality and the final on‐couch dose calculation is performed on the HyperSight CBCT using Acuros iCBCT reconstruction, the CT does not need to cover the entire region to be treated.

An important aspect of the OSCAR workflow regarding the reference image preparation concerns the definition of the simulation isocenter (user origin) within the Ethos software (i.e., Ethos Treatment Management V3.0 and Ethos Treatment Planning V2.0), which has to be assigned to an anatomically unambiguous and reproducible landmark. When a dCT without reference markers is used, careful selection of such a landmark is essential to ensure reliable patient setup. Reproducible landmarks can be palpated in many anatomical regions or identified externally; common examples include the upper or lower edge of the sternum, the pubic bone, and, for cranial treatments, external reference points such as the outer canthus in combination with the patient midline. Since longitudinal movement of the Ethos couch is limited (−17 cm to +30 cm^10^), the user origin must be placed near the planned treatment isocenter. Failure to do so may result in geometrical constraints during on‐couch adaptation.

In cases where for the OSCAR workflow no CT dataset was available and only MRI imaging existed, an institutional alternative approach was applied: A total‐body CT provided by Varian Medical Affairs^11^ was imported into a generic patient in ARIA (V18.0), and all OARs required for the OSCAR workflow were contoured on this CT. This dataset represents a non‐patient‐specific anatomical reference. For actual patients, the total‐body CT was exported in anonymized form, with all DICOM identifiers removed and subsequently re‐imported into ARIA. The full scan length was used in all cases to prevent potential errors of an incorrect CT dataset or missing CT slices in later workflow steps. The CT could then be imported into the Ethos software and registered with secondary datasets such as MRI. As the most relevant OARs are automatically contoured by Ethos, the only manual step is the delineation of at least one CTV. Owing to partial integration between ARIA and Ethos, contouring can be performed in ARIA, after which the resulting structures can be assigned in Ethos. Irrespective of the workflow used, image registration must be validated within the Ethos software. This alternative workflow represents an institutional solution for exceptional cases, was applied under local clinical governance procedures, and did not replace patient‐specific imaging for final dose calculation.

### 2.4 RT intent selection and field configuration

To streamline and standardize the OSCAR workflow, planning directive (RT intent) templates were created for each anatomical region and fractionation scheme listed in Table 1. In the Ethos software, the RT Intent is a framework that unifies the prescription, dose‐fractionation scheme, and prioritized clinical objectives within a single planning template, including target and OAR definitions. It automatically drives plan optimization during both initial planning and on‐couch adaptive planning. Each template contains all relevant RT intent information, including the clinical target volume (CTV) and planning target volume (PTV), with the PTV defined as a derived structure from the CTV using a 5 mm isotropic margin. The templates also include the most relevant OARs for the respective anatomical site contained in Table 1 and phase‐specific parameters, such as plan normalization and the interval between two sessions, fixed at D50% = 100% for the PTV and 6 h, respectively. Additionally, each template contains optimization objectives for target volumes and OARs, with the primary focus on achieving uniform dose coverage of the PTV. All RT intent parameters remain modifiable; for instance, a larger CTV‐to‐PTV margin may be applied when clinically necessary. To utilize the Ethos influencer structures (i.e., OARs defined by Varian that may affect the shape and position of the target volume) effectively, RT intents for the anatomical sites mediastinum and craniospinal regions were selected for thoracic and brain treatments, respectively. In these RT intents, the heart (for the thoracic region) and the spinal canal and brain (for the craniospinal region) serve as influencers.^12^ For all other anatomical sites listed in Table 1, the relevant section of the spine with the spinal canal as the influencer was used.

After the RT intent is authorized, a 9‐field equidistant IMRT plan was consistently selected for the OSCAR workflow, as this configuration provided clinically acceptable plan quality while maintaining reasonable optimization times. The plan underwent clinical review and approval by both a physician and a physicist before the online adaptive workflow could begin. To enable subsequent on‐couch PSQA, the approved plan was also exported to secondary dose calculation software (Mobius3D, V4.0.2).

### 2.5 Online adaptive session and QA

The OSCAR workflow does not differ substantially from the conventional on‐couch adaptive workflow. However, two critical aspects must be considered: First, the patient must be positioned at the anatomical landmark defined as simulation isocenter in the Ethos software. Second, because CBCT image quality is crucial, the treatment volume must be fully captured within the CBCT field of view. Consequently, the X‐ and Y‐blades of the kV tube must be fully opened, and increasing the mAs is recommended to enhance contrast. Since the HU‐to‐density curve was implemented for 125 kV only and to mitigate errors, an additional CBCT preset was created to automatically select the appropriate tube voltage, maximum mAs, and Acuros iCBCT reconstruction algorithm. It should be emphasized that for longitudinally extended target volumes the *extended CBCT* mode has to be selected. In this mode, two kV‐CBCT images acquired 14 cm apart in the longitudinal axis are automatically merged into a 38.1 cm long CBCT image.^10^

The subsequent procedure mirrors the conventional Ethos online adaptive workflow: influencers and OARs are automatically contoured and reviewed by a physician, the target volume is propagated onto the CBCT and manually corrected, if necessary, the plan is optimized, and a secondary dose calculation using Mobius3D is performed. Following clinical and technical approval, an additional CBCT may be acquired to adjust for translational shifts prior to irradiation.

### 2.6 Strategies for subsequent fractions

After treatment, the quality of the CBCT should be carefully evaluated for each individual patient and clinical situation to determine whether it is sufficient for precise target volume definition. Challenges may arise, for example, when high soft tissue contrast is required for gross tumor volume (GTV) delineation, in patients with increased adiposity, or in re‐irradiation scenarios.

Following the first adaptive session, several options exist for continuing treatment with additional treatment fractions to reach the prescribed total dose. First, the online adaptive workflow can be continued, particularly if interfractional anatomical changes are expected and if the patient is compliant. Second, the approved target volumes and OARs can be used to generate a conventional IGRT VMAT plan based on HyperSight CBCT and corresponding structure set, enabling faster delivery at the linac. Third, the irradiated static gantry IMRT plan can be re‐imported into the Ethos‐software and approved for further IGRT treatment, if the plan quality is sufficient. Fourth, if the CBCT image quality is deemed insufficient for reliable target definition, a dedicated planning CT can be acquired immediately after the first fraction. The decision on how to proceed must be made collaboratively by the physician, physicist, and radiation therapist (RTT).

### 2.7 Safety considerations and task assignment for the OSCAR workflow

Before the OSCAR workflow can be initiated, several patient safety considerations must be addressed, and responsibilities must be clearly assigned to the physician, physicist, and RTT. Once the physician has confirmed the indication for urgent palliative treatment initiation, the fractionation scheme must be defined and documented in writing by the physician in the clinic's Record and Verify (R&V) system.

Furthermore, for each anatomical site and each fractionation scheme listed in Table 1, an RT intent template must be created in advance to reduce the risk of workflow‐related errors. As indicated in Section 2.4, this template must include all relevant OARs for the anatomical site to be treated, the target volumes to be contoured, and the associated objectives for plan optimization. This should be performed jointly by a physician and a physicist to address both clinical and technical aspects. In addition, all phase properties of the RT intent must be defined.

The treatment plan generated on the reference image within the Ethos software using the created RT intent must be carefully reviewed by both a physician and a physicist according to the four‐eye principle. In this way, the relevant treatment parameters, including the dose per fraction, target volume definition, and assignment of the user origin, can be verified.

Since on‐couch secondary dose calculation using Mobius is only possible if the reference plan has been exported to Mobius in advance, this must be verified by the physicist before treatment begins. During the online adaptive session, the patient is positioned on couch and aligned to the lasers according to the assigned user origin by the RTT. For the acquired HyperSight CBCT, it is highly recommended that a dedicated CBCT preset for the OSCAR workflow is established, including the correct tube voltage, maximum mAs, and Acuros iCBCT reconstruction algorithm. The selection of the correct CBCT preset should be verified by both the physician and the physicist before the CBCT is acquired by the RTT in the online adaptive session.

**FIGURE 2:**
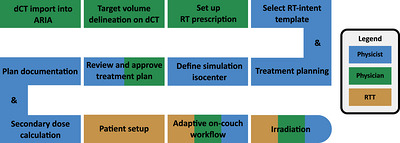
Schematic overview of the OSCAR workflow, illustrating key steps from reference imaging and RT intent creation to on‐couch adaptation and treatment delivery. The color coding indicates the professional group responsible for each task.

Because considerable time pressure exists once the HyperSight CBCT has been acquired, all steps during the online adaptive session should be performed by the physician and physicist according to the four‐eye principle. This includes assessing the influencers and other OARs for completeness and accuracy, evaluating target volume delineation, reviewing plan quality, and assessing the associated QA via secondary dose calculation. The documentation of the workflow, including the assignment of responsibilities to the physician, medical physicist, and RTT, is strongly recommended. Figure 2 provides a schematic overview of the OSCAR workflow and illustrates the tasks assigned to each professional group using color coding.

### 2.8 Dosimetric comparison IMRT versus VMAT

Within the RT intent template selected for the OSCAR workflow, optimization objectives were defined for target volumes and OARs, with the primary aim of achieving uniform dose coverage of the PTV. Because the most relevant OARs^12^ were automatically contoured during the adaptive session, they were directly incorporated into plan optimization. The Intelligent Optimization Engine (IOE) within the Ethos software was used for automated plan generation. Previous studies^13^, ^14^ have shown that the IOE can produce clinically acceptable static gantry IMRT treatment plans. For the first 11 patients, the automatically generated 9‐field IMRT plans from the OSCAR workflow were dosimetrically compared to VMAT plans generated in Eclipse V18.0. VMAT plans were optimized by experienced physicists using institutional planning standards.

Since multiple fractionation schemes were clinically prescribed, relative dose values normalized to the prescribed dose were used for comparison. Both IMRT and VMAT dose distributions were calculated on the HyperSight CBCT acquired during the adaptive session, using contours approved by the physician during online adaptation. Given the limited patient cohort and the heterogeneity of treated anatomical sites and fractionation schemes, no formal inferential statistical testing was performed. The dosimetric evaluation was intended to be descriptive in nature.

## 3 RESULTS

### 3.1 Workflow feasibility and timing

The OSCAR workflow was successfully established for the first 11 patients for whom rapid initiation of radiotherapy was clinically indicated according to the decision tree shown in Figure 1. The individual workflow steps as well as the involved medical staff depicted in Figure 2 and the corresponding time requirements are summarized in Table 2. It should be noted that the specified time values were not derived from a statistical analysis; rather they provide a general overview based on our current clinical experience and were estimated through an interdisciplinary assessment.

**TABLE 2:** Overview of individual workflow steps of the OSCAR workflow, associated personnel, and approximate time requirements. It should be noted that the reported time values represent estimated workflow durations and were not derived from a statistical analysis.

Workflow step	Estimated time and staff responsible for OSCAR workflow
dCT export into ARIA	5 min (physician)
Target volume delineation on dCT	20 min (physician)
Set up RT prescription	5 min (physician)
Select RT intent template & Treatment planning	15 min (physicist)
Define simulation isocenter	5 min (physicist)
Review and approve treatment plan	30 min (physician & physicist)
Plan documentation & Secondary dose calculation	10 min (physicist)
Patient setup	5 min (RTT)
Adaptive on‐couch workflow	10 min (physician, physicist, RTT)
Irradiation	5 min (physician, physicist, RTT)
Total time	**1 h 50 min**

Overall, the total time required to initiate treatment was approximately 1 h and 50 min, which is in good agreement with the findings reported in the FAST‐METS study,^2^ Malkov et al.^15^ or by Price et al. ^16^ Compared with a conventional CT‐simulation‐based palliative radiotherapy workflow, the average time a patient spends in the center is approximately 5 h.^3^, ^17^

The adaptive treatment fraction could be completed in approximately 20 min, including patient setup, the adaptive workflow, and irradiation. This duration was found to be acceptable even for patients experiencing pain or limited ability to remain immobilized. We further observed that the more accurately the target volume is defined on the underlying CT dataset for generating the reference plan, the more reliably the on‐couch target volume propagation works, thereby reducing the need for manual corrections and further decreasing the time a patient spends on the couch.

### 3.2 Patient cohort and treatment concepts

Table 3 summarizes the treated anatomical regions, prescribed fractionation schemes, and subsequent treatment strategies for all patients treated using the OSCAR workflow. All patients received their first fraction using the OSCAR workflow.

**TABLE 3:** Summary of patients treated using the OSCAR workflow, including anatomical region, prescribed fractionation scheme, and subsequent treatment strategy.

Patient No. (#)	Entity	Concept	Further treatment
1	Lumbar vertebral body & sacrum	10 × 3 Gy	Adaptive treatment only
2	Pulmonary metastasis & vertebral body involvement	1 × 8 Gy	Adaptive treatment only
3	Whole brain radiation with orbital infiltration	10 × 3 Gy	VMAT for 9 remaining fractions using HyperSight CBCT
4	Pelvic vertebral body & soft tissue infiltration	10 × 3 Gy	VMAT for 9 remaining fractions using HyperSight CBCT
5	Sacrum & anterior bladder wall	5 × 4 Gy	VMAT for 4 remaining fractions using HyperSight‐CBCT
6	Pelvic vertebral body & soft tissue infiltration	5 × 4 Gy	VMAT for 4 remaining fractions using HyperSight CBCT
7	Lumbar vertebral body & sacrum	5 × 4 Gy	VMAT for 4 remaining fractions using HyperSight CBCT
8	SCLC	10 × 3 Gy	VMAT for 9 remaining fractions using HyperSight CBCT
9	Acute cervical bleeding	10 × 3 Gy	VMAT for 9 remaining fractions using HyperSight CBCT
10	Pulmonary venous congestion	3 × 3 Gy	VMAT for 2 remaining fractions using HyperSight CBCT
11	Whole brain radiation	10 × 3 Gy	VMAT for 9 remaining fractions using HyperSight CBCT

Except for patient 3, all patients received at least one dCT prior to treatment, which was used to set up the RT intent within the Ethos software. For patient 3, MRI was the only available imaging modality; therefore, the institutional alternative workflow described in Section 2.3 using a universal whole‐body CT dataset was applied. Patients 1 and 2 received all prescribed fractions using the adaptive OSCAR workflow only. For all remaining patients, an Eclipse‐based VMAT plan was generated on the HyperSight CBCT and corresponding structure set acquired during the adaptive session and was used for subsequent fractions following standard clinical approval and PSQA. To allow a comprehensive dosimetric comparison, VMAT plans were also generated for patients 1 and 2.

### 3.3 Dosimetric comparison of adaptive IMRT and VMAT

**FIGURE 3:**
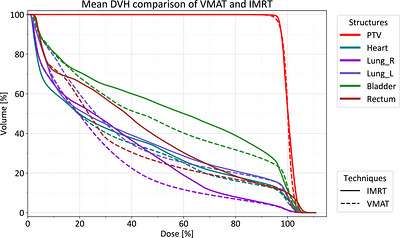
Mean DVH comparison between adaptive 9‐field IMRT plans generated within the OSCAR workflow (solid lines) and corresponding VMAT plans optimized in Eclipse (dashed lines), with dose values normalized to the prescribed dose.

Because multiple treatment sites with different fractionation schemes and, consequently, different OARs were clinically involved, a relative dose‐volume histogram (DVH) comparison of the adaptive 9‐field‐IMRT and the associated VMAT plan was performed. The mean DVH curves for all patients are shown in Figure 3, and the corresponding quantitative analysis is summarized in Table 4.

**TABLE 4:** Quantitative comparison of selected DVH parameters for adaptive 9‐field IMRT plans generated within the OSCAR workflow and corresponding VMAT plans, with dose values and standard deviations expressed relative to the prescribed dose.

Dosimetric Parameter	Number of occurences	IMRT dose values (%)	VMAT dose values (%)
PTV D_98%_	11	96.42 ± 0.80	95.4 ± 1.5
PTV D_50%_	11	100.15 ± 0.18	100.07 ± 0.10
PTV D_2%_	11	104.48 ± 0.51	103.94 ± 0.76
PTV Homogeneity Index (D_2%_—D_98%_)/D_50%_	11	8.05 ± 0.56	8.5 ± 2.1
Heart D_Mean_	5	36 ± 30	35 ± 28
Lung_R D_Mean_	5	32 ± 16	27 ± 12
Lung_L D_Mean_	5	38 ± 25	39 ± 23
Bladder D_Mean_	4	56 ± 33	50 ± 31
Rectum D_Mean_	4	43 ± 25	35 ± 26

Both IMRT and VMAT plans fulfilled clinical acceptance criteria for target coverage and OAR sparing. While PTV coverage was comparable between both techniques, with similar homogeneity and conformity, VMAT plans demonstrated improved sparing for several OARs. It should be noted that not only two different treatment techniques but also two distinct optimization algorithms were compared, namely the user‐driven Eclipse Photon Optimizer (PO) and the Ethos IOE. In Eclipse, achieving a treatment plan with adequate target coverage while maintaining OAR sparing requires a considerable amount of time due to iterative manual inverse planning. In contrast, the automated Ethos IOE is designed to reduce planning effort by optimizing the trade‐off between plan quality and planning efficiency. This behavior is also described by Pokharel et al.^13^ and Sait et al.^18^

## 4 DISCUSSION

In this work, we report our initial clinical experience with the OSCAR workflow in a cohort of 11 patients. By using a dCT, or even a universal whole‐body CT dataset, to generate the reference plan, we demonstrated that treatment can be initiated within a short timeframe of approximately 2 h. Because numerous workflow steps must be completed within a short period, patient safety must be ensured, and potential sources of error must be eliminated from the outset. Therefore, the implementation of OSCAR requires not only technical feasibility but also a structured workflow with clear role definitions and standardized decision pathways. This requires, for example, a robust RT intent template with objectives capable of generating clinically acceptable treatment plans. Particular attention must be given to prevent both underdosage and overdosage of the target volume. Since OARs may overlap with the PTV, the corresponding OAR objectives should be selected conservatively; overly stringent objectives may produce sharp dose gradients adjacent to these structures, thereby increasing fluence modulation complexity. Moreover, since the anatomical CTV may be located close to the patient's skin, a 5 mm inner ring of the body contour is subtracted from the resulting PTV. This approach is intended to mitigate potentially high‐fluence segments due to the build up effect at the skin, which could lead to overdosage in the event of even minor patient movement. Accurate anatomical contouring is therefore essential to ensure complete coverage of the clinically relevant region within the CTV. Additional parameters that must be defined within the template for the RT intent include the minimum time interval between two consecutive treatments, the selection of adaptive treatment, and the plan normalization. In our clinic, these values were set to 6 h and D50% = 100%, respectively.

Using one of our RT intent templates resulted in a clinically acceptable 9‐field IMRT plan. Because the on‐couch optimization time for VMAT is considerably longer than for static gantry IMRT,^13^ VMAT is currently not suitable for online adaptation, particularly for patients who experience severe pain or have difficulty remaining still for extended periods. Pokharel et al.^13^ report average optimization times of 2.5, 5, and 13 min to generate a 9‐field IMRT, 12‐field IMRT, and dual‐arc VMAT plans, respectively. Furthermore, for Ethos IOE it has been shown^13^, ^19^, ^20^ that the quality of VMAT plans is not inherently superior to that of static gantry IMRT plans. If, in the future, both the optimization time and VMAT plan quality can be further improved in the Ethos software, VMAT may become a viable option for on‐couch adaptation to further reduce the time patients must remain on the treatment couch. To already leverage both main advantages of a VMAT plan, namely shorter treatment times and improved sparing of individual OARs,^21^ a VMAT plan was created in Eclipse based on the HyperSight CBCT and the corresponding structure set of the adaptive session. For this newly generated treatment plan, the standard radiotherapy process chain was carried out, including clinical plan review, documentation, and plan‐specific QA. This hybrid approach preserved the benefit of immediate first‐fraction delivery while allowing subsequent fractions to be delivered more efficiently.

As demonstrated in Section 3, if the quality of the adapted static gantry IMRT plan is considered clinically acceptable, treatment may also be continued using this IMRT plan within a conventional IGRT workflow. Because the delivered IMRT plan is automatically transferred to ARIA for treatment documentation, it can be assigned to the acquired HyperSight CBCT and the corresponding structure set. In addition, within the Ethos software, the RT intent must be modified such that adaptive treatment is deselected and the acquired HyperSight CBCT, together with its associated structures, is selected as the planning CT instead of the diagnostic CT. After the user origin has been set correctly, the RT intent can be authorized again, and the adapted IMRT plan can be imported and approved for subsequent IGRT treatments. This approach allows the workflow to be further streamlined, enabling prompt delivery of the second fraction.

Because there is no reference point on the underlying CT dataset, an anatomically unambiguous location must be selected for patient positioning. As noted above, this reference point must be located near the planned treatment isocenter due to the limited longitudinal travel of the treatment couch.^10^ If an Eclipse‐VMAT plan is used for the remaining fractions, the selected reference point must be marked on the HyperSight CBCT within Eclipse. We observed that only the imaging isocenter is annotated on the CBCT and is automatically adopted as the user origin after the on‐couch session. Consequently, this reference point must be adjusted manually so that the applied delta couch shift is taken into account so the final reference point coincides with the anatomical reference location on the patient. The applied delta couch shift can be retrieved from the reference plan report in the Ethos software. In the case of an extended CBCT, the reference point lies between both MV‐isocenters. Standardized instructions for RTTs and physicists, including clear documentation of the anatomical reference point and applied couch shifts, were therefore considered essential for safe reproducibility of the OSCAR workflow.

Despite the demonstrated feasibility and efficiency of the OSCAR workflow, several limitations must be considered. The first aspect is the time pressure experienced once the patient is already positioned on the treatment couch. From a risk management perspective, a separate and structured execution of workflow steps, as in the conventional process, allows for more targeted preparation and multiple opportunities to identify potential sources of error without the constraints of immediate time pressure. As indicated in Section 2, it is essential that parameters within the RT intent, including phase properties, considered OARs, and optimization objectives for both OARs and target volumes, are carefully selected to mitigate errors from the outset and to ensure robustness in generating clinically acceptable treatment plans. To enhance safety and to reduce the risk of errors, workflow steps should be performed according to a four‐eye principle. Second, our evaluation is limited by the small patient cohort and heterogeneity of treated indications and anatomical sites. While this reflects the real‐world nature of urgent radiotherapy, it limits the ability to generalize dosimetric findings or derive statistical conclusions. A further limitation is that additional time at the linac is required as steps typically performed during CT simulation must now be conducted at the treatment unit. This includes, for example, forming a thermoplastic mask,^22^ which may pose practical challenges when incorporated directly at the linac. Nonetheless, this disadvantage is compensated by the significant benefit of enabling immediate treatment initiation for patients requiring urgent therapy.

Several organizational and procedural aspects must be addressed at the institutional level prior to implementation of the OSCAR workflow. General workflow considerations for CBCT‐based oART have been described by Schiff et al.^23^ After sufficient experience has been gained with oART, the OSCAR workflow should be rehearsed under realistic conditions using an anthropomorphic phantom within an interdisciplinary team comprising physicians, medical physicists, and RTTs. To further mitigate potential sources of error, detailed written instructions should be developed and clearly assigned to the respective professional groups. Experiences gained from early clinical cases should be promptly integrated into these instructions and incorporated as a permanent component of an interdisciplinary training program. Such simulation‐based rehearsals, combined with comprehensive written documentation, have been shown to uncover latent workflow vulnerabilities and to enhance interdisciplinary team performance.^24^, ^25^

In summary, the OSCAR workflow enhances patient comfort because no planning CT is required, thereby shortening overall time to first fraction and allowing treatment to begin more rapidly. At the same time, unnecessary travel and transport can be avoided. A recent study by Wurschi et al.^26^ clearly demonstrated the extent to which costs and CO_2_ emissions can be reduced when travel or transport to the clinic is minimized. Consequently, both the time patients and their accompanying persons spent in the clinic as well as the need for medical staff support can be reduced. These considerations are becoming increasingly important in light of workforce shortages, rising transport costs, and the present concern of climate change. While these aspects were not quantified in the present work, they represent relevant secondary benefits of simulation‐free workflows and should be addressed in future analyses. Prospective data collection with predefined safety, workflow, and clinical outcome metrics will be essential to systematically evaluate these aspects and to support broader clinical adoption of the OSCAR workflow.

## 5 CONCLUSION

In conclusion, the OSCAR workflow represents a novel and clinically feasible approach to initiate radiotherapy without the need for a dedicated planning CT. The workflow was established on a limited initial patient cohort with heterogeneous treatment sites, and its applicability will be expanded as clinical experience is gained. We have demonstrated that clinically acceptable treatment plans could be generated within a short timeframe by utilizing robust RT intent templates with predefined optimization objectives, enabling rapid delivery of the first fraction in urgent and palliative indications. Further studies will be required to determine whether the OSCAR workflow can be extended to additional disease sites and fractionation schemes, including ultra‐hypofractionated regimens, and to evaluate clinical outcomes, safety aspects, and resource utilization in larger patient cohorts.

## AUTHOR CONTRIBUTIONS

Robert Schindhelm, Anne Richter and Gary Razinskas designed the study and did most of the analysis. All authors discussed the results, reviewed the manuscript and agree with publication.

## CONFLICT OF INTEREST STATEMENT

The authors declare no potential conflicts of interest.
